# Improving the practicality of using non-aversive handling methods to reduce background stress and anxiety in laboratory mice

**DOI:** 10.1038/s41598-019-56860-7

**Published:** 2019-12-30

**Authors:** Kelly Gouveia, Jane L. Hurst

**Affiliations:** 1 Mammalian Behaviour and Evolution Group, Institute of Integrative Biology, University of Liverpool, Leahurst Campus, Neston, CH64 7TE UK; 2 Present Address: Department of Biological Sciences, University of Chester, Chester, CH1 4BJ UK

**Keywords:** Cardiovascular models, Cancer models, Biological models, Gastrointestinal models, Genetic models

## Abstract

Handling can stimulate stress and anxiety in laboratory animals that negatively impacts welfare and introduces a confounding factor in many areas of research. Picking up mice by the tail is a major source of handling stress that results in strong aversion to the handler, while mice familiarised with being picked up in a tunnel or cupped on the open hand show low stress and anxiety, and actively seek interaction with their handlers. Here we investigate the duration and frequency of handling required for effective familiarisation with these non-aversive handling methods, and test whether this is sufficient to prevent aversion and anxiety when animals then experience immobilisation and a mild procedure (subcutaneous injection). Very brief handling (2 s) was sufficient to familiarise mice with tunnel handling, even when experienced only during cage cleaning. Brief but more frequent handling was needed for familiarisation with cup handling, while pick up by tail induced strong aversion even when handling was brief and infrequent. Experience of repeated immobilisation and subcutaneous injection did not reverse the positive effects of tunnel handling. Our findings demonstrate that replacing tail with tunnel handling during routine cage cleaning and procedures provides a major refinement with little if any cost for familiarisation.

## Introduction

Animals managed in captivity can be highly sensitive to interaction with humans, particularly if they associate this with negative experiences, resulting in acute and chronic stress responses, anxiety, reduced productivity and animals that are harder to handle e.g.^1,2^. This is particularly a problem among laboratory animals, where stress and anxiety in response to human contact (or the threat of human contact) negatively impacts animal welfare, increases the difficulty of handling animals that may attempt to bite or escape, and is a potential confounding factor in many scientific experiments^3,4^. As handling is an unavoidable and variable procedure in managing captive animals, this type of confounding factor is very difficult to control.

To minimise these problems, animals need to be familiarised with handling to allow them to learn that contact with humans is not itself harmful^5–7^. However, Hurst & West^8^ showed that the method used to handle mice has a major impact on whether or not familiarisation with handling leads to a substantial reduction in aversion towards the handler, the stress experienced during handling, and the anxiety this induces. Picking mice up in a handling tunnel, or cupping them on the open hand, leads to substantial voluntary interaction with the handler and reduced stress and anxiety^8^. However, mice respond negatively to being picked up by the base of the tail and do not readily habituate to this widely used method. Subsequent studies have confirmed that these strong differences between handling methods generalise across laboratories, handlers and different strains of mice^9–14^. Further, compared to mice picked up by tail, mice handled by non-aversive tunnel or cupping methods have reduced plasma corticosterone, reduced blood glucose and improved glucose tolerance^10^; reduced glomerular lesions in a glomerulonephritis model^15^; improved reliability in behavioural tests that require exploration of test stimuli^11^; improved responsiveness to sucrose reward^12^; and are easier to handle^13^. Mice handled using a tunnel also show reduced pain grimace scores compared to tail-handled mice^16^, show less fearful behaviour and continue to interact readily with their handler even after experiencing procedures such as oral gavage, intraperitoneal injection, tattooing or ear-tagging^13,16^. Thus, there is substantial evidence that using these non-aversive handling methods in place of picking up mice by the tail provides a substantial benefit for mouse welfare, while potentially also increasing the ease of handling for researchers and carers. Further, as background stress and anxiety in laboratory animals can be important confounding factors in many areas of scientific research, minimising these responses can also significantly reduce variability^13^ and improve the reliability of test responses^11,12^.

Studies typically use extended handling periods to familiarise animals to handling e.g.^17–20^. Hurst & West^8^ familiarised animals by picking them up and holding them by their assigned method for 60 s per day over a two week period. Subsequent investigations into the impact of these methods have followed generally similar handling schedules to the original study. However, prolonged handling of multiple animals introduces a substantial time cost. This may be a price that researchers are willing to pay to improve the reliability of animal responses in specific experiments that are highly sensitive to handling effects; but applying this approach more generally to all animals in a facility that may contain many hundreds of rodents is not likely to be feasible or, at least, very costly. Now we know that familiarising mice to non-aversive handling methods has such a profound effect on their response to human contact, it is essential to understand how long animals need to be handled for effective familiarisation, and how frequently, so that time costs can be minimised. We also need to understand the impact of brief handling during routine husbandry procedures, such as cage cleaning. Recently, we found substantial differences in anxiety and novelty investigation between mice that were familiarised with brief tail, tunnel or cup handling just during fortnightly cage cleans prior to being tested in a novel arena^11^. Those handled by non-aversive tunnel or cup methods showed much more reliable responses in an habituation-dishabituation behavioural test than those picked up by tail. This suggests that brief handling at cage cleaning might be sufficient to familiarise mice with these non-aversive methods (or alternatively to sensitise mice to aversive tail handling). If this is the case, implementation of non-aversive handling during routine husbandry across facilities could provide an effective and practical way to minimise handling effects with little if any time cost. This would provide a major refinement to animal welfare and minimise a confounding factor that influences many areas of scientific research.

In contrast to the traditional tail method, using a tunnel or cupping mice on the open hand does not involve direct physical restraint of the animal, but instead moves the surface on which the animal stands (tunnel or hand). However, restraint is necessary for many common procedures, such as dosing or blood sampling, or when checking some aspects of animal health. Surprisingly, mice familiarised with tunnel handling or cupping but not with restraint did not reduce their willingness to interact with a handler after experiencing complete immobilisation by scruff restraint^8^. Tunnel handled mice also continued to show substantially greater voluntary interaction compared to tail handled mice after repeated daily oral gavage^13^ and after identity marking by tattoo or ear-tagging which induce some pain^16^. In each of these studies, though, mice were familiarised with handling that was more prolonged than they would normally experience during routine husbandry. As many animals are likely to experience such procedures when used in the laboratory, we need to understand whether less extensive handling familiarisation is sufficient to reduce handling-related stress and benefit welfare, even when animals may associate handling with experience of physical restraint and common mild procedures.

To establish the most practical but effective implementation of non-aversive handling, here we investigate the impact of hold duration when animals are picked up (Experiment 1), and the frequency of handling (Experiment 2), on the response of mice to tunnel or cup handling compared to tail handling. We also test response to repeated immobilisation when restrained by the scruff (Experiment 1), or to repeated immobilisation and subcutaneous injection (Experiment 3), after mice have been familiarised with brief handling that can be carried out quickly or during routine background husbandry. We show that the brief handling required to transfer mice between cages (c. 2 s) is sufficient to familiarise mice with tunnel handling, even when this is only experienced at fortnightly cage cleans. Brief but more frequent handling is required to familiarise animals with being cupped on the open hand, but mice picked up by tail show strong aversion and anxiety even when handled briefly and infrequently. Tunnel handled mice continue to interact just as readily with a handler after repeated immobilisation and subcutaneous injection and show low anxiety, while tail handled mice show as much aversion and anxiety when just picked up by the tail as those immobilised and injected. The requirement for only very brief familiarisation with tunnel handling makes this a highly practicable method for avoiding handling stress in the laboratory, while picking up mice by the tail should be avoided as far as possible, even for short lifts.

## Results

### Effects of hold duration and handling method

To examine the influence of how long animals are held after being picked up by different methods, 7–8 week old C57BL/6 mice living in single-sex pairs were handled daily for 5 days using one of three methods (tail, tunnel or cup) and held for 2, 10, 30 or 60 s on each occasion. Those held for 60 s were picked up twice and held for 30 s each time to replicate the original Hurst & West study^8^. Mice picked up by tail were supported on the handler’s sleeve while being held for 10, 30 or 60 s, but those picked up for only 2 s were unsupported in line with widespread practice for brief lifts of mice^6,21^. We assessed the willingness of mice to interact voluntarily with the handler immediately before and after the first and fifth daily handling periods^8^. We also assessed anxiety in an open field test^22–25^ when mice were picked up and transferred to the arena by their assigned method two days after the last daily handling session (see Experiment 1 Methods for full details, and Supplementary Table 1 for a summary of experimental design).

**Figure 1:**
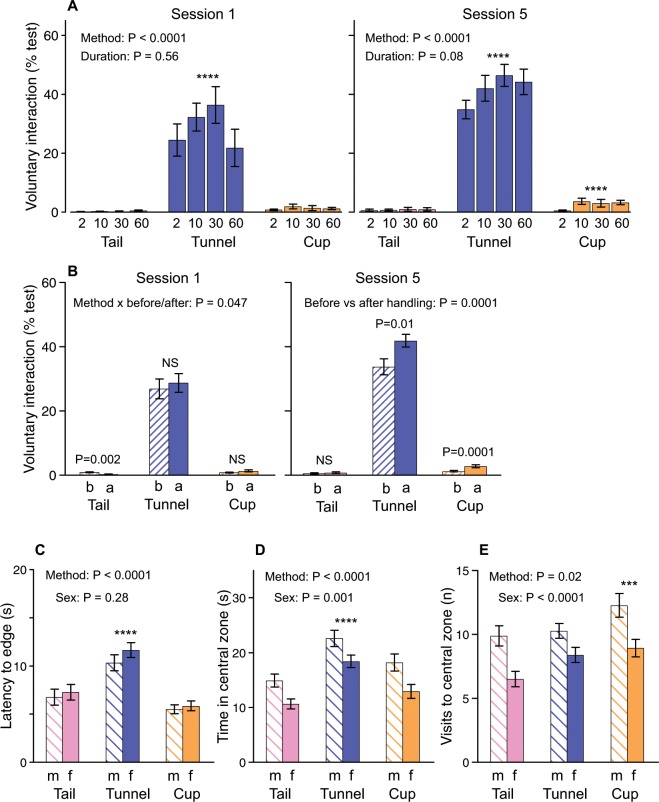
Effect of handling method and hold duration on voluntary interaction and anxiety (mean ± sem). Duration of voluntary interaction with the handling device (hand for tail and cup, or hand holding tunnel) (**A**) after the first and fifth daily handling session according to hold duration and method, and (**B**) immediately before (b) and after (a) handling in the first and fifth sessions pooled across hold durations. (**C**,**D**,**E**) Measures of anxiety in an open field test conducted two days after the fifth handling session (m: males, f: females). P values from univariate (**A**) or repeated measures ANOVAs (**B,C**) shown in Tables 1 and 2. Asterisks indicate significance of planned comparisons between tunnel or cup versus tail (*P < 0.05, **P < 0.01, ***P < 0.005, ****P < 0.001). Data for tunnel and cup handled mice pooled across groups experiencing same or tail handling at cage cleaning prior to the experiment. Number of mice/cages: Tail = 16/8 m + 16/8 f (8/4 mf per duration), Tunnel = 32/16 m + 32/16 f (16/8 mf per duration), Cup = 32/16 m + 32/16 f (16/8 mf per duration).

Hold duration had no significant effects on the willingness of mice to interact with the handler after either the first or fifth handling session, though after the fifth handling session mice held for only 2 s by any method showed a non-significant tendency (P = 0.08) to interact slightly less with the handler than those held for longer (Fig. 1A, Table 1). Notably, hold duration did not influence the willingness of mice picked up by tail to interact, indicating that mice find even very short lifts by tail aversive. In strong contrast, the method of handling had large and highly significant effects on voluntary interaction. Mice picked up in a tunnel showed substantially greater interaction than those picked up by tail even after their first experience, rising to an even greater difference between tunnel and tail by session 5, consistent with previous findings^8,9,11–13^. Mice cupped on the open hand also showed significantly more voluntary interaction than those picked up by tail by their fifth experience (P < 0.0001), but the increase in interaction in this case was relatively small. Change in response immediately before versus after handling also differed according to handling method (Fig. 1B, Table 1): mice picked up by non-aversive tunnel or cup methods were more willing to interact with the handler immediately after their experience of handling than before (session 5), but those picked up by tail showed minimal (session 5) or even less willingness to interact afterwards than before (session 1).

**Table 1:** Effects of handling method and hold duration on voluntary interaction with the handler.

Voluntary interaction	First handling session		Fifth handling session	
**(a) Response after handling**				
Handling method	F_2,52_ = 138.4	**P < 0.0001**	F_2,56_ = 331.8	**P < 0.0001**
Hold duration	F_3,52_ = 0.69	P = 0.56	F_3,56_ = 2.38	P = 0.08
Sex	F_1,52_ = 0.24	P = 0.63	F_1,56_ = 2.59	P = 0.11
Method × duration	F_6,52_ = 0.59	P = 0.74	F_6,56_ = 0.77	P = 0.60
Method × sex	F_2,52_ = 1.78	P = 0.18	F_2,56_ = 1.82	P = 0.17
Duration × sex	F_3,52_ = 0.21	P = 0.89	F_3,56_ = 0.72	P = 0.55
3 way interaction	F_6,52_ = 0.40	P = 0.88	F_6,56_ = 0.61	P = 0.72
**Planned contrasts:**				
tail vs tunnel		**P < 0.0001**		**P < 0.0001**
tail vs cup		P = 0.07		**P < 0.0001**
**(b) Immediately before vs after handling**				
Handling method	F_2,52_ = 159.0	**P < 0.0001**	F_2,56_ = 402.0	**P < 0.0001**
Hold duration	F_3,52_ = 0.47	P = 0.71	F_3,56_ = 1.62	P = 0.19
Sex	F_1,52_ = 0.01	P = 0.98	F_1,56_ = 2.51	P = 0.12
Before vs after handling	F_1,52_ = 0.42	P = 0.52	F_1,56_ = 16.85	**P = 0.0001**
Method × duration	F_6,52_ = 0.23	P = 0.96	F_6,56_ = 0.84	P = 0.54
Method × before/after	F_2,52_ = 3.25	**P = 0.047**	F_2,56_ = 2.05	P = 0.14
Duration × before/after	F_3,52_ = 0.81	P = 0.50	F_3,56_ = 1.79	P = 0.16
Sex × before after	F_1,52_ = 1.06	P = 0.31	F_1,56_ = 0.08	P = 0.78
**Before vs after handling:**				
tail	F_1,8_ = 19.41	**P = 0.002**	F_1,8_ = 1.34	P = 0.34
tunnel	F_1,24_ = 0.69	P = 0.42	F_1,24_ = 7.04	**P = 0.014**
cup	F_1,24_ = 0.74	P = 0.40	F_1,24_ = 20.24	**P = 0.0001**
**Planned contrasts:**				
tail vs tunnel		**P < 0.0001**		**P < 0.0001**
tail vs cup		P = 0.39		**P = 0.001**

Mice were picked up by their assigned method (tail, tunnel or cup) and held for 2, 10, 30 or 60 s per day each day for five days. Voluntary interaction was measured before and after handling on days 1 and 5, averaged for both mice tested together in the same cage (data shown in Fig. 1A,B and Supplementary File 1). Data were log transformed to meet assumptions for parametric analysis. **(a)** Univariate ANOVA with planned contrasts between tail and non-aversive methods. **(b)** Repeated measures ANOVA comparing response before and after handling (higher order interactions not shown, but all were non-significant). Significant effects (P < 0.05) are in bold. Sample size for the first handling session was reduced because of technical problems with 4 video files, though all animals underwent their assigned handling.

Anxiety in the open field test was significantly influenced by handling method. Mice handled by tunnel were slower to leave the central zone after release than those picked up by tail (Fig. 1C). They also spent more time in the central zone during the test (Fig. 1D) as visits to the central zone were more prolonged than for tail handled mice (Table 2), consistent with lower anxiety. Cupped mice entered the central zone more frequently than those picked up by tail (Fig. 1E) but failed to spend more time there after 5 days of cupping versus tail handling, so the duration per visit was lower than for mice picked up by the tail (Table 2). Hold duration had a small effect on the frequency of visits to the central zone (Table 2); mice held for only 2 s made fewer central zone visits (7.92 ± 0.54) than those familiar with more prolonged handling (10.15 ± 0.38), but there was no significant effect on total time in the central zone (Table 2).

**Table 2:** Effects of handling method and hold duration on anxiety responses in an open field test.

	Time in central zone (s)		Visits to central zone (n)		Time per central zone visit (s)		Latency to arena edge (s)		Stretched attend (n)	
Handling method	F_2,54_ = 15.62	**P < 0.0001**	F_2,54_ = 4.54	**P = 0.015**	F_2,54_ = 22.88	**P < 0.0001**	F_2,54_ = 25.01	**P < 0.0001**	F_2,54_ = 0.35	P = 0.71
Handling duration	F_3,54_ = 2.14	P = 0.11	F_3,54_ = 3.29	**P = 0.027**	F_3,54_ = 0.34	P = 0.80	F_3,54_ = 1.14	P = 0.34	F_3,54_ = 2.00	P = 0.13
Sex	F_1,54_ = 15.85	**P = 0.0002**	F_1,54_ = 20.50	**P < 0.0001**	F_1,54_ = 0.47	P = 0.50	F_1,54_ = 1.21	P = 0.28	F_1,54_ = 0.002	P = 0.96
Method × duration	F_6,54_ = 0.93	P = 0.49	F_6,54_ = 0.64	P = 0.70	F_6,54_ = 0.68	P = 0.67	F_6,54_ = 0.54	P = 0.84	F_6,54_ = 1.54	P = 0.18
Method × sex	F_2,54_ = 0.44	P = 0.65	F_2,54_ = 0.37	P = 0.69	F_2,54_ = 0.25	P = 0.78	F_2,54_ = 0.24	P = 0.79	F_2,54_ = 0.97	P = 0.38
Duration × sex	F_3,54_ = 1.39	P = 0.26	F_3,54_ = 0.68	P = 0.57	F_3,54_ = 1.38	P = 0.26	F_3,54_ = 0.51	P = 0.68	F_3,54_ = 0.43	P = 0.74
3 way interaction	F_6,54_ = 0.78	P = 0.59	F_6,54_ = 1.72	P = 0.13	F_6,54_ = 0.85	P = 0.54	F_6,54_ = 0.81	P = 0.57	F_6,54_ = 1.20	P = 0.32
**Planned contrasts:**										
tail vs tunnel		**P < 0.0001**		P = 0.18		**P < 0.0001**		**P < 0.0001**		P = 0.85
tail vs cup		P = 0.18		**P = 0.005**		**P = 0.038**		P = 0.14		P = 0.47

Mice had previously been picked up by their assigned method and held for different durations (2, 10, 30 or 60 s) for 5 daily handling sessions. Mice were delivered to the centre of the open field arena using their assigned method (data shown in Fig. 1C–E and Supplementary File 1). Repeated measures ANOVAs, taking into account two mice per cage as a within-subjects factor, with planned contrasts between tail and non-aversive tunnel or cup methods (time in central zone and time per visit log transformed to meet assumption of parametric analysis). Significant effects (P < 0.05) are in bold.

### Scruff restraint and handling method

Tunnel and cup handling methods involve moving the platform (tunnel or hand) that mice are standing on without direct physical restraint of the animal, but restraint is often necessary for health checks and other procedures. Hurst & West^8^ reported that immobilising mice through scruff restraint did not reverse the taming effects of these non-aversive handling methods, but measured voluntary interaction only after a single restraint event and did not assess effects in an established test of anxiety. As an extension to Experiment 1, we therefore tested the voluntary interaction of mice immediately before and after experience of daily scruff restraint, repeated for 3 days once mice had been familiarised with 5 days of handling by tail, tunnel or cup (see above). We assessed voluntary interaction a further 24 h after the third restraint to look for any longer term effects as well as testing anxiety in an elevated plus maze on the following day (see Experiment 1 Methods for full details, and Supplementary Table 1 for a summary of experimental design).

**Figure 2:**
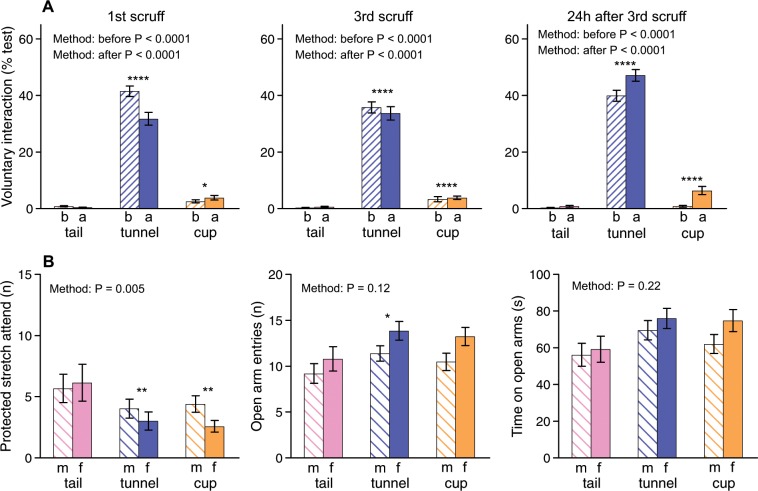
Effect of handling method and restraint by scruff on voluntary interaction and anxiety (mean ± sem). (**A)** Duration of voluntary interaction with handling device (hand for tail and cup, or hand holding tunnel) assessed immediately before (b) and after (a) scruff restraint among mice familiarised for one week with tail, tunnel or cup methods in first and third daily restraint sessions, and when picked up but not restrained 24 h later. **(B)** Measures of anxiety in an elevated plus maze test conducted two days after experience of 3^rd^ scruff restraint among males (m) and females (f) familiarised with tail, tunnel or cup handling. Data for mice held for different durations during handling familiarisation pooled. P values from repeated measures ANOVAs shown in Tables 3 and 4. Asterisks indicate significance of planned comparisons between tunnel or cup versus tail (*P < 0.05, **P < 0.01, ***P < 0.005, ****P < 0.001). Data for tunnel and cup handled mice pooled across groups experiencing same or tail handling at cage cleaning prior to the experiment (N sizes as for Fig. 1).

Repeated immobilisation through scruff restraint did not reverse the taming effects of non-aversive handling methods. Mice picked up by tunnel or cup showed substantially more voluntary interaction with their handler than those picked up by tail both immediately before and after scruff restraint, even when this was repeated (Fig. 2A; Table 3). The difference between tunnel and tail handled mice was particularly marked, even though tunnel handled mice previously had very little experience of restraint while those picked up by tail experienced some physical restraint each time they were handled. Cup handled mice also showed significantly more interaction than tail handled mice before and particularly after scruff restraint, although voluntary interaction was much less than for tunnel handled mice (Fig. 2A, Table 3). Nonetheless, cup handled mice consistently showed improved interaction with the handler immediately following scruff restraint compared to before restraint, which was not seen among tail handled mice. Before testing the response of mice to scruff restraint, they had been familiarised with 5 days of handling by tail, tunnel or cup and held for 2, 10, 30 or 60 s each time they were picked up (see previous section). The duration of handling that mice had previously experienced before scruff restraint had no effects on their immediate response to this immobilisation. The longer-term response measured 24 h after repeated immobilisation suggested a small effect of prior hold duration that differed between handling methods, as mice picked up by cupping for only 2 s showed reduced interaction compared to those held on the hand for longer. However, this was due to previous experience of handling rather than a differential response to scruff restraint as the same low interaction was evident prior to scruffing (Fig. 1A).

**Table 3:** Effects of scruff restraint and prior handling on voluntary interaction with the handler.

Voluntary interaction	First scruff		Third scruff		24 h after 3^rd^ scruff	
**(a) Before scruff restraint**						
Handling method	F_2,56_ = 220.1	**P < 0.0001**	F_2,56_ = 175.9	**P < 0.0001**	F_2,55_ = 247.02	**P < 0.0001**
Prior hold duration	F_3,56_ = 1.28	P = 0.29	F_3,56_ = 2.63	P = 0.06	F_3,55_ = 2.35	P = 0.082
Sex	F_1,56_ = 0.70	P = 0.41	F_1,56_ = 2.05	P = 0.16	F_1,55_ = 1.21	P = 0.28
Method × duration	F_6,56_ = 0.44	P = 0.85	F_6,56_ = 1.95	P = 0.09	F_6,55_ = 1.50	P = 0.20
Method × sex	F_2,56_ = 0.13	P = 0.88	F_2,56_ = 0.52	P = 0.60	F_2,55_ = 0.63	P = 0.54
Duration × sex	F_3,56_ = 0.46	P = 0.72	F_3,56_ = 0.05	P = 0.98	F_3,55_ = 0.46	P = 0.71
3 way interaction	F_6,56_ = 0.68	P = 0.67	F_6,56_ = 0.19	P = 0.98	F_6,55_ = 2.00	P = 0.08
**Planned contrasts:**						
tail vs tunnel		**P < 0.0001**		**P < 0.0001**		**P < 0.0001**
tail vs cup		**P = 0.017**		**P < 0.0001**		**P < 0.0001**
**(b) After scruff restraint**						
Handling method	F_2,56_ = 117.1	**P < 0.0001**	F_2,56_ = 122.3	**P < 0.0001**	F_2,55_ = 208.84	**P < 0.0001**
Prior hold duration	F_3,56_ = 2.04	P = 0.12	F_3,56_ = 2.54	P = 0.07	F_3,55_ = 4.90	**P = 0.004**
Sex	F_1,56_ = 4.16	**P = 0.046**	F_1,56_ = 1.82	P = 0.18	F_1,55_ = 0.08	P = 0.78
Method × duration	F_6,56_ = 1.42	P = 0.22	F_6,56_ = 1.58	P = 0.17	F_6,55_ = 2.69	**P = 0.023**^†^
Method × sex	F_2,56_ = 0.54	P = 0.59	F_2,56_ = 0.28	P = 0.76	F_2,55_ = 0.05	P = 0.95
Duration × sex	F_3,56_ = 0.25	P = 0.86	F_3,56_ = 0.65	P = 0.59	F_3,55_ = 0.91	P = 0.44
3 way interaction	F_6,56_ = 0.25	P = 0.96	F_6,56_ = 1.55	P = 0.18	F_6,55_ = 1.73	P = 0.13
**Planned contrasts:**						
tail vs tunnel		**P < 0.0005**		**P < 0.0005**		**P < 0.0001**
tail vs cup		**P < 0.0005**		**P < 0.0005**		**P < 0.0001**
**(c) Before vs after restraint**						
Before vs after	F_1,56_ = 2.97	P = 0.09	F_1,56_ = 2.22	P = 0.14	F_1,55_ = 21.02	**P < 0.0001**
Method × before/after	F_2,56_ = 7.25	**P = 0.002**	F_2,56_ = 4.49	**P = 0.016**	F_2,55_ = 2.79	P = 0.07
Tail	F_1,8 = _5.43	**P = 0.048**	F_1,8 = _1.62	P = 0.24	F_1,8 = _1.93	P = 0.20
Tunnel	F_1,24_ = 9.64	**P = 0.005**	F_1,24_ = 2.67	P = 0.11	F_1,24_ = 10.90	**P = 0.003**
Cup	Interaction with sex		F1,24 = 5.76	**P = 0.025**	F1,24 = 15.56	**P = 0.001**
Cup: males	F_1,12_ = 0.70	P = 0.42				
Cup: females	F_1,12_ = 16.97	**P = 0.001**				

During the week prior to testing, mice had been picked by their assigned method (tail, tunnel, cup) and held for 2, 10, 30 or 60 s per day (prior hold duration). Mice were then picked up by their assigned method prior to 10 s scruff restraint on three successive days. Voluntary interaction, averaged for both mice tested together in the same cage, was assessed immediately before and after scruff restraint on the first and third day, and 24 h later, before and after pick up by the assigned method for 2 s without scruff restraint (data shown in Fig. 2A). Data were log transformed to meet assumptions of parametric analysis. **(a**,**b)** Univariate and **(c)** repeated measures ANOVAs with planned contrasts between tail and non-aversive methods. Significant effects (P < 0.05) are in bold. Repeated measures ANOVA (**c**) included prior hold duration and sex but these were not significant. Response to first scruff among those handled by cupping depended on sex, with females showing significantly greater interaction after scruffing than before. ^†^Significant interaction between handling method and prior hold duration 24 h after 3^rd^ scruff was because cupped mice showed lowest interaction when previously held for 2 s and highest interaction when held for 60 s per day (Bonferoni post-hoc comparison, P = 0.002) while prior hold duration did not influence those handled by tail or tunnel.

After repeated scruff restraint, mice picked up by the tail showed a higher frequency of protected stretch attend postures in the elevated plus maze compared to both tunnel and cup handled mice. This is a measure of anxiety and risk assessment^26,27^, where mice stretch forwards onto the open arms of the maze from the protection of the walled arms but then withdraw without entering the arm. Tunnel but not cup handled mice also entered the open arms more often than those picked up by tail, but greater time on the open arms did not reach statistical significance (Fig. 2B; Table 4). There were no differences in the time or frequency on closed arms (Table 4).

**Table 4:** Effect of prior handling on anxiety in an elevated plus maze following repeated scruff restraint.

	Protected stretch attend (n)		Entry to open arms (n)		Time on open arms (s)		Entry to closed arms (n)		Time on closed arms (s)	
Handling method	F_2,32_ = 6.24	**P = 0.005**	F_2,32_ = 2.32	P = 0.12	F_2,32_ = 1.61	P = 0.22	F_2,32_ = 0.07	P = 0.94	F_2,32_ = 0.45	P = 0.64
Prior hold duration	F_3,32_ = 2.73	P = 0.06	F_3,32_ = 0.96	P = 0.42	F_3,32_ = 0.50	P = 0.69	F_3,32_ = 0.24	P = 0.87	F_3,32_ = 1.27	P = 0.30
Sex	F_1,32_ = 0.09	P = 0.77	F_1,32_ = 4.15	**P = 0.050**	F_1,32_ = 0.74	P = 0.40	F_1,32_ = 3.34	P = 0.08	F_1,32_ = 0.01	P = 0.94
Method × duration	F_6,32_ = 2.36	P = 0.053	F_6,32_ = 0.47	P = 0.83	F_6,32_ = 0.42	P = 0.86	F_6,32_ = 0.21	P = 0.97	F_6,32_ = 0.70	P = 0.65
Method × sex	F_2,32_ = 2.61	P = 0.09	F_2,32_ = 0.30	P = 0.75	F_2,32_ = 0.34	P = 0.72	F_2,32_ = 0.84	P = 0.44	F_2,32_ = 0.49	P = 0.62
Duration × Sex	F_3,32_ = 0.39	P = 0.76	F_3,32_ = 0.50	P = 0.79	F_3,32_ = 0.61	P = 0.62	F_3,32_ = 1.14	P = 0.35	F_3,32_ = 0.60	P = 0.62
3 way interaction	F_6,32_ = 0.72	P = 0.63	F_6,32_ = 0.95	P = 0.47	F_6,32_ = 0.55	P = 0.77	F_6,32_ = 0.95	P = 0.47	F_6,32_ = 0.64	P = 0.70
Planned contrasts:										
tail vs tunnel		**P = 0.010**		**P = 0.044**		P = 0.09		P = 0.73		P = 0.53
tail vs cup		**P = 0.009**		P = 0.15		P = 0.23		P = 0.56		P = 0.97

Mice had been familiarized with their assigned handling method (tail, tunnel or cup) for 5 days when held for 2, 10, 30 or 60 s per day (prior hold duration), then restrained by the scruff for 10 s on three successive days before the elevated plus maze test (data for measures of anxiety (protected stretch attend, entry and time on open arms) shown in Fig. 2B). Repeated measures ANOVAs took into account two mice per cage as a within-subjects factor, with planned contrasts between tail and non-aversive tunnel or cup methods. Batch was also included in analyses as mice in the first batch tested showed consistently higher measures of anxiety compared to those in the second batch, but with the same pattern of response to handling method. Significant effects (P =  < 0.05) are in bold.

### Frequency of handling

Our first experiment showed that hold duration had little impact on the response of mice to the handler or anxiety, and mice could be familiarised with non-aversive tunnel handling through brief pick up (2 s), equivalent to that experienced during cage cleaning. However, mice were handled daily to replicate the frequent handling typically used to familiarise animals with handling procedures immediately prior to an experiment. In Experiment 2, we investigated whether brief handling to transfer mice between cages only at a fortnightly cage clean was sufficient frequency to familiarise mice and show positive responses to tunnel handling. We also investigated whether more frequent but only very brief handling could improve the response to cup handling. For this experiment, BALB/c mice were randomly assigned to handling method and frequency on arrival in our unit aged 3–4 weeks. Single-sex pairs were cleaned at fortnightly intervals using their assigned method to transfer between cages, with voluntary interaction with the handler assessed after the first and fourth cage cleans. Before the fifth cage clean, mice in half of the cages were briefly handled on a daily basis for nine sessions (c. 2 s, equivalent to a cage transfer), while the other cages were again handled only at the fifth cage clean when voluntary interaction was again tested. We then assessed the anxiety of all mice in an elevated plus maze test (see Experiment 2 Methods for full details, and Supplementary Table 1 for a summary of experimental design).

**Figure 3:**
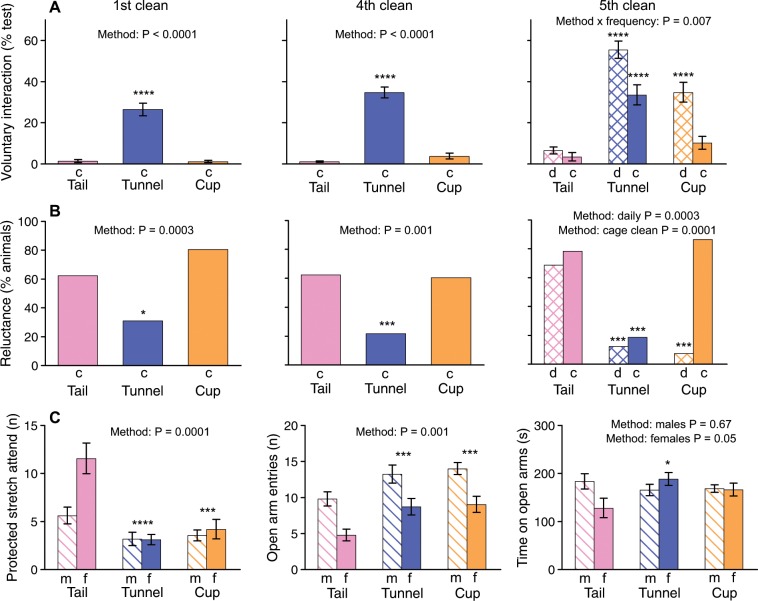
Effect of handling method and frequency on willingness to interact with handler and anxiety. Mice were handled briefly only at cleaning for the first 4 fortnightly cage cleans. Between 4^th^ and 5^th^ cage clean, mice received supplementary brief (2 s) daily handling for nine sessions (d) or continued only to be handled at cage cleaning (c). **(A)** Duration of voluntary interaction with the handling device (hand for tail and cup, or hand holding tunnel) assessed immediately after cage cleaning (mean ± sem). **(B)** Percentage of mice reluctant to be handled when approached by the handler, measured immediately following post-handling voluntary interaction test. **(C)** Measures of anxiety in an elevated plus maze test conducted two days following the fifth cage clean (m: males, f: females). P values from univariate (A, Table 5) or repeated measures (C, Table 6) ANOVAs, or contingency chi-squared tests (**B**). Asterisks indicate significance of planned comparisons between tunnel or cup versus tail (*P < 0.05, **P < 0.01, ***P < 0.005, ****P < 0.001). N sizes for mice/cages: Tail = 16/8 m + 16/8 f, Tunnel = 16/8 m + 16/8 f, Cup = 16/8 m + 16/8 f (half handled daily (d) and half at caged clean only (c) following 4^th^ cage clean).

After four cage cleans, voluntary interaction differed substantially between handling methods. Mice picked up and transferred between cages in a tunnel spent much longer interacting with the handling device held unmoving in the cage compared to those picked up and transferred by tail. However, those picked up briefly by cupping showed no more voluntary interaction than those picked up by tail (Fig. 3A; Table 5). This was not very surprising as mice had also shown relatively little interaction after 5 days of brief cup handling compared to tunnel handling in our first experiment, though they were a little more willing to interact than those handled by tail. Hurst & West^8^ found that willingness to interact after cup handling rose sharply in the second week of daily 60 s cup handling. The addition of brief daily pick up (2 s) for half the cages before the 5^th^ cage clean dramatically improved interaction among cup handled but not tail handled mice (Fig. 3A; Table 5). This additional daily experience increased interaction by tunnel handled mice too, but those picked up in a tunnel were much more willing to interact than tail handled mice whether they experienced handling additional to cage cleaning or not (Fig. 3A). As voluntary interaction was assessed at cage cleaning in this experiment, behaviour immediately after handling was assessed once animals had been transferred to a clean cage but willingness to interact immediately before this was assessed in the animals’ familiar home cage. Notably, before the 5^th^ transfer to a clean cage, mice that experienced additional daily cup handling spent just as much time in voluntary interaction as those given additional daily tunnel handling (percentage of test time for cup: 60.2 ± 5.2; tunnel: 63.9 ± 2.8; tail: 22.6 ± 5.2; F_2,18_ = 29.0, P < 0.0001). However, after transfer to a clean cage, tunnel handled mice maintained a higher level of interaction than cup handled mice (Fig. 3A), suggesting that mice familiarised with tunnel handling were more robust to the disturbance induced by cage transfer.

**Table 5:** Effects of handling method and frequency on voluntary interaction with the handler.

Voluntary interaction	1st cage clean		4^th^ cage clean		5^th^ cage clean	
**Response after handling**						
Handling method	F_2,36_ = 54.72	**P < 0.0001**	F_2,36_ = 92.76	**P < 0.0001**	F_2,36_ = 65.33	**P < 0.0001**
Frequency	F_1,36_ = 0.05	P = 0.83	F_1,36_ = 0.10	P = 0.75	F_1,36_ = 34.25	**P < 0.0001**
Sex	F_1,36_ = 0.24	P = 0.63	F_1,36_ = 0.04	P = 0.85	F_1,36_ = 0.14	P = 0.71
Method × frequency	F_2,36_ = 0.04	P = 0.97	F_2,36_ = 0.34	P = 0.71	F_2,36_ = 5.73	**P = 0.007**
Method × sex	F_2,36_ = 0.18	P = 0.84	F_2,36_ = 0.28	P = 0.76	F_2,36_ = 4.03	**P = 0.026**
Frequency × sex	F_1,36_ = 1.25	P = 0.27	F_1,36_ = 0.04	P = 0.84	F_1,36_ = 0.08	P = 0.78
3 way interaction	F_2,36_ = 0.57	P = 0.57	F_2,36_ = 0.03	P = 0.97	F_2,36_ = 1.78	P = 0.18
**Planned contrasts:**						
tail vs tunnel		**P < 0.0001**		**P < 0.0001**		**P < 0.0001**
tail vs cup		P = 0.93		P = 0.34		**P < 0.0001**
**Daily vs cage clean only**^**†**^						
tail					F_1,12_ = 1.30	P = 0.28
tunnel					F_1,12_ = 12.12	**P = 0.005**
cup					F_1,12_ = 24.47	**P = 0.0003**
**Male response after handling**^**‡**^						
Handling method					F_2,18_ = 47.76	**P < 0.0001**
Frequency					F_1,18_ = 16.27	**P = 0.001**
Method × frequency					F_2,18_ = 2.29	P = 0.13
**Planned contrasts:**						
tail vs tunnel						**P < 0.0001**
tail vs cup						**P = 0.010**
**Female response after handling**^**‡**^						
Handling method					F_2,18_ = 22.79	**P < 0.0001**
Frequency					F_1,18_ = 17.99	**P < 0.0001**
Method × frequency					F_2,18_ = 5.08	**P = 0.018**
**Planned contrasts:**						
tail vs tunnel						**P < 0.0001**
tail vs cup						**P = 0.001**

Mice were picked up briefly by their assigned method (tail, tunnel or cup) to transfer them between cages at four fortnightly cage cleans; between the 4^th^ and 5^th^ cage clean, half were assigned to brief daily handling (approx. 2 s) while the other half were only handled at cage cleaning. Voluntary interaction, averaged for both mice tested together in the same cage, tested immediately after mice were transferred to a clean cage (data shown in Fig. 3A). Univariate ANOVAs after 1^st^, 4^th^ and 5^th^ cage clean with planned contrasts between tail and non-aversive tunnel or cup methods. Significant effects (P < 0.05) are in bold. ^†^Daily vs fortnightly handling was assessed separately for each handling method due to a significant interaction between handling method and frequency at 5^th^ cage clean. ^‡^Male and female response assessed separately due to a significant interaction between handling method and sex at 5^th^ cage clean. A significant interaction between handling method and frequency among females was due to much greater voluntary interaction among females handled daily by cupping between 4^th^ and 5^th^ cage clean compared to those handled only at cage cleaning.

As attempts to run away from the handler increase the time and skill required to handle animals, we used a further test to assess whether mice ran away from the hand or avoided entering the tunnel when the handler approached to pick them up (see Methods Experiment 2 for full details of the handling reluctance test). Each mouse was tested individually in an empty home cage, after testing voluntary interaction following cage cleaning. When handled only at cage cleaning, most tail and cup handled mice attempted to avoid handling while avoidance was significantly less among those picked up in a tunnel (Fig. 3B). Supplementary brief handling overcame attempts by animals cupped on the open hand to run away, while most of those picked up by tail continued to run away from the hand (Fig. 3B).

Handling method influenced behaviour in an elevated plus maze test conducted after handling familiarisation. Both tunnel and cup handled mice were more active in moving around the maze than those picked up by tail, scoring significantly more entries to both open and closed arms (Fig. 3C; Table 6). By contrast, tail handled mice (particularly females) spent more time in the central hub of the maze where they showed a much higher frequency of protected stretch attend – stretching out onto the open arms of the maze but then withdrawing (Fig. 3C; Table 6), a characteristic of anxiety^28,29^. Females picked up by tunnel also spent more time on the open arms than those picked up by tail, though this difference was not significant among males or for either sex handled by cupping (Fig. 3C; Table 6). Increasing handling frequency between cage cleans did not further reduce anxiety measures compared to those handled only at cage cleaning prior to the test (Table 6).

**Table 6:** Effects of handling method and frequency on anxiety in an elevated plus maze test.

	Protected stretch attend		Entry to open arms (n)		Time on open arms (s)		Entry to closed arms (n)		Time in central hub (s)	
Handling method	F_2,31_ = 11.93	**P = 0.0001**	F_2,31_ = 8.30	**P = 0.001**	F_2,31_ = 0.74	P = 0.49	F_2,31_ = 7.04	**P = 0.003**	F_2,31_ = 3.86	**P = 0.03**
Frequency	F_1,31_ = 0.01	P = 0.95	F_1,31_ = 0.46	P = 0.50	F_1,31_ = 1.73	P = 0.20	F_1,31_ = 2.57	P = 0.12	F_1,31_ = 1.62	P = 0.21
Sex	F_1,31_ = 5.22	**P = 0.03**	F_1,31_ = 17.91	**P = 0.0002**	F_1,31_ = 0.95	P = 0.34	F_1,31_ = 10.21	**P = 0.003**	F_1,31_ = 3.74	P = 0.06
Method × frequency	F_2,31_ = 0.26	P = 0.77	F_2,31_ = 0.25	P = 0.78	F_2,31_ = 0.52	P = 0.60	F_2,31_ = 0.12	P = 0.89	F_2,31_ = 0.55	P = 0.58
Method × sex	F_2,31_ = 3.46	**P = 0.04**	F_2,31_ = 0.14	P = 0.87	F_2,31_ = 3.12	P = 0.058	F_2,31_ = 0.65	P = 0.53	F_2,31_ = 2.65	P = 0.09
Frequency × sex	F_1,31_ = 0.75	P = 0.39	F_1,31_ = 0.12	P = 0.73	F_1,31_ = 0.21	P = 0.65	F_1,31_ = 0.20	P = 0.66	F_1,31_ = 0.74	P = 0.40
3 way interaction	F_2,31_ = 0.54	P = 0.59	F_2,31_ = 0.02	P = 0.98	F_2,31_ = 2.41	P = 0.11	F_2,31_ = 0.16	P = 0.85	F_2,31_ = 1.68	P = 0.20
**Planned contrasts:**										
tail vs tunnel		**P < 0.0001**		**P = 0.002**		P = 0.23		**P = 0.013**		**P = 0.024**
tail vs cup		**P = 0.001**		**P = 0.001**		P = 0.54		**P = 0.001**		**P = 0.025**
**Handling method**										
Males only	F_2,16_ = 2.22	P = 0.14			F_2,16_ = 0.41	P = 0.67				
tail vs tunnel		P = 0.07				P = 0.42				
tail vs cup		P = 0.13				P = 0.49				
Females only	F_2,15_ = 9.62	**P = 0.002**			F_2,15_ = 3.66	**P = 0.05**				
tail vs tunnel		**P = 0.001**				**P = 0.02**				
tail vs cup		**P = 0.004**				P = 0.10				

Mice were handled by their assigned method (tail, tunnel or cup) to transfer them to a clean cage at five fortnightly cage cleans. Half also received brief daily handling between the 4^th^ and 5^th^ cage cleans. Mice were delivered to the elevated plus maze test by their assigned method on the day after their 5^th^ cage clean (data for measures of anxiety (protected stretch attend, entry and time on open arms) shown in Fig. 3C). Repeated measures ANOVAs took into account two mice per cage as a within-subjects factor, with planned contrasts between tail and tunnel or cup methods. Significant effects (P =  < 0.05) are in bold. The effect of handling method was examined for each sex separately where there was a significant or almost significant interaction between handling method and sex.

### Experience of subcutaneous injection does not reverse effects of non-aversive handling

Lastly, in Experiment 3, we tested whether experience of subcutaneous injection would reverse the taming effects of non-aversive handling, as an example of a mild invasive regulated procedure that is presumed to induce brief pain and discomfort^30,31^. Naïve BALB/c females were randomly assigned to brief handling by either tail or tunnel. To familiarise them with being picked up, they were handled by their assigned method at cage cleaning and also picked up daily for approximately 2 s over 10 sessions before the main experiment began. For each handling method, half of the cages were assigned to immobilisation by scruff restraint and subcutaneous injection of saline, while the other half experienced only a brief control pick up by their assigned method (full details in Methods Experiment 3, and Supplementary Table 1 for a summary of experimental design; note that response to scruff restraint without injection was addressed in Experiment 1 so was not repeated here).

**Figure 4:**
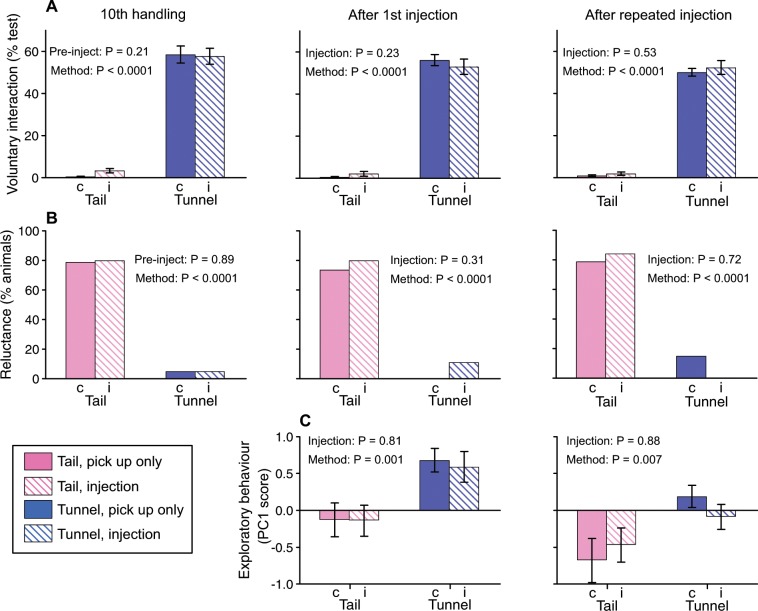
Response of mice familiarised with tunnel or tail handling to subcutaneous injection. Mice were familiarised with brief (2 s) pick up by tunnel or tail in 10 daily sessions. They were then either picked up, restrained by scruff and given a subcutaneous injection (i) or just picked up briefly without restraint or injection (c). **(A)** Duration of voluntary interaction with the handling device (hand for tail and cup, or hand holding tunnel) assessed immediately after 10^th^ handling familiarisation, 1^st^ injection or control pick up, and after 5^th^ injection or control pick up (mean ± sem). Pre-inject: for the 10^th^ handling familiarisation, mice assigned to the injection treatment are compared to those in the control pick up treatment, although neither group had experienced injection at this point. **(B)** Percentage of mice reluctant to be handled when approached by the handler. **(C)** Exploratory (positive score) versus anxious behaviour (negative score) in a modified open field test conducted after 5^th^ injection or control pick up (mean ± sem). P values from Mann-Whitney (A, Table 7), repeated measures ANOVA (C, Table 8), or contingency chi-squared tests (**B**). N = 20 females/10 cages per treatment group.

Prior to experience of subcutaneous injection, tunnel handled mice spent much more time in voluntary interaction with the handler (Fig. 4A) and were much less likely to try to avoid being picked up than those handled by tail (Fig. 4B), as expected from previous experiments. The same differences between handling methods were evident when tested one week after immobilisation and injection (Fig. 4A,B; Table 7). Notably, experience of injection did not influence either the willingness to interact with the handler or reluctance to be handled. To test whether repeated experience of injection induced a stronger effect, mice received four further daily injections before interaction with the handler was tested again. There was still no difference in voluntary interaction between mice that experienced repeated restraint and injection compared to those picked up only briefly by their assigned method, and response did not change between first and repeated injection / pick up (Fig. 4A,B; Table 7). Tunnel-handled mice continued to interact much more with the handler than those picked up by tail and showed much less reluctance to be handled, with no change in willingness to interact in response to the experience of restraint and injection (Fig. 4A,B). Mice picked up by tail showed even lower voluntary interaction following experience of injection than before (Table 7), but interaction was just as low among control mice picked up by the tail that did not experience restraint and injection (Fig. 4A). Thus, overall, willingness to interact with the handler was influenced by the method used to pick mice up but not by experience of immobilisation and injection.

**Table 7:** Effects of subcutaneous injection and prior handling by tail or tunnel on voluntary interaction with the handler.

Voluntary interaction	Before 1^st^ injection		After 1^st^ injection		After 5^th^ injection	
Handling method	z = 5.41	**P < 0.0001**	z = 5.34	**P < 0.0001**	z = 5.41	**P < 0.0001**
Injection vs control pick up	z = 1.27	P = 0.21	z = 0.23	P = 0.84	z = 0.53	P = 0.53
**Comparison between time points**						
Tail, control pick up	χ^2^ = 1.42	P = 0.49				
Tail, injection	χ^2^ = 8.60	**P = 0.014**				
Tunnel, control pick up	χ^2^ = 2.60	P = 0.27				
Tunnel, injection	χ^2^ = 0.67	P = 0.72				

Mice were picked up briefly by their assigned handling method (tail or tunnel) for 10 days prior to treatment. Half the mice were then assigned to either control pick up by their assigned method or pick up, scruff restraint and subcutaneous injection, repeated on 5 days. Voluntary interaction was assessed immediately before and after handling in the 10^th^ handling session, 1 week after first subcutaneous injection or control pick up, and one week after 5^th^ subcutaneous injection or control pick up. The proportion of test time interacting voluntarily with the handler was averaged for tests conducted immediately before and after handling by the assigned handling method at each time point (data shown in Fig. 4A). Mann-Whitney tests assessed differences in averaged voluntary interaction between handling methods and between injection versus control pick up at each time point. Repeated measures nonparametric Friedman tests compared response across the 3 time points for each treatment group. Only mice picked up by tail showed significantly reduced interaction following experience of injection. Significant effects (P < 0.05) are in bold.

We also assessed anxiety in a modified open field test carried out immediately following experience of immobilisation plus injection or control pick up (tested after first and after repeated experience). Mice were placed in the periphery of a clean open field with a streak of male mouse urine on a tile in the centre of the arena to stimulate investigation of a novel attractive scent, similar to the set up used by Gouveia & Hurst^11^. Anxious animals avoid open areas and show reduced exploration and investigation of rewarding stimuli. As each of the behaviours measured in the open field were strongly correlated, we derived a single component of behaviour by principal components analysis that explained 43% of variance in the test; more positive scores reflected high exploration while more negative scores reflected anxiety (positive weighting to number of lines crossed, frequency of visits to central urine tile and time sniffing urine; negative weighting to latency to first visit central zone and frequency of stretch attend postures, Table 8). Experience of injection had no effect on anxiety while handling method had a highly significant effect (Fig. 4C; Table 8). Mice picked up by tunnel showed reduced anxiety compared to those picked up by tail, regardless of injection experience. Although investigation of the urine stimulus reduced in the second test after repeated experience while latency to enter the central zone increased, this was the case whether mice experienced repeated injection or just a control pick up, with tail-handled mice continuing to show more anxious behaviour than tunnel handled mice (Fig. 4C; Table 8).

**Table 8:** Effects of subcutaneous injection and prior handling by tail or tunnel on exploratory behaviour in a modified open field test.

PC1 score		
**After 1**^**st**^ **and 5**^**th**^ **injection**		
Handling method	F_1,36_ = 14.03	**P = 0.001**
Injection vs control pick up	F_1,36_ = 0.05	P = 0.83
Repetition (first vs multiple)	F_1,36_ = 27.55	**P < 0.001**
Method × injection	F_1,36_ = 0.60	P = 0.44
Method × repetition	F_1,36_ = 0.52	P = 0.48
Injection × repetition	F_1,36_ = 0.01	P = 0.93
3 way interaction	F_1,36_ = 1.02	P = 0.32
**After first injection**		
Handling method	F_1,36_ = 14.20	**P = 0.001**
Injection vs control pick up	F_1,36_ = 0.06	P = 0.81
Method × injection	F_1,36_ = 0.05	P = 0.83
**After 5**^**th**^ **injection**		
Handling method	F_1,36_ = 8.34	**P = 0.007**
Injection vs control pick up	F_1,36_ = 0.02	P = 0.88
Method × injection	F_1,36_ = 1.26	P = 0.27

Mice were picked up briefly by their assigned handling method (tail or tunnel) for 10 days prior to treatment. Mice then experienced either control pick up by their assigned method, or pick up, scruff restraint and subcutaneous injection, repeated 5 times. Anxiety in a modified open field test was assessed immediately after first and fifth experience of injection or control pick up. Principal components analysis derived a single component that explained 43% of variance across all trials. This contrasted the total number of line crosses (weighted 0.68), frequency of visits to urine tile (0.80) and time sniffing stimulus urine (0.61) with latency to first enter the central zone (−0.75) and frequency of stretch attend postures (−0.35), such that positive scores reflected high exploratory behaviour and low anxiety (data shown in Fig. 4C). Repeated measures ANOVA examined consistency of behaviour after single or repeated treatment while univariate ANOVA examined each time point separately. Significant effects (P < 0.05) are in bold.

## Discussion

Our study confirms that laboratory mice picked up by tail show strong aversion and higher anxiety compared to those handled by non-aversive tunnel and cup methods, as reported in previous studies^8–16^. Going further, we have shown that these differences in behaviour are evident even when mice are lifted up only briefly, as experienced when animals are transferred between cages for example. Perhaps surprisingly, our study has also revealed that the method used to pick up mice has a substantial effect on aversion and anxiety while immobilisation through scruff restraint and experience of subcutaneous injections did not. Indeed, we found that immobilisation and injection had no effect on these sensitive behavioural measures beyond the experience of being picked up in the first place, and these procedures did not reduce the positive impact of tunnel handling for avoiding handling stress.

Lengthy and frequent handling is normally advised to increase ease of handling among laboratory animals and reduce stress and anxiety in response to human contact^6,32^. However, the duration for which animals are held once picked up had very little effect on handling-induced aversion or anxiety within the 2–60 s range that we studied. Voluntary interaction tended to be lowest after the briefest 2 s pick up across methods, and mice picked up by cupping showed improved interaction following 10 s scruff restraint if they had been familiarised with being held on the hand for longer than 2 s. Nonetheless, the method used to pick mice up was far more important for inducing a positive response to handling familiarisation than the duration of handling. Notably, mice picked up and held only very briefly by the tail showed just as much avoidance of the hand compared to those experiencing more prolonged tail restraint. Thus, the negative responses to tail handling reported in previous studies that have used restraint by the tail of up to 60 s are due to the experience of being picked up by the tail, not to how long they were restrained by the tail. Likewise, previous research has shown that mice habituated to being held on the experimenter’s hand following pick up by the tail still exhibit high anxiety on behavioural testing, which demonstrates that further contact with the hand or having body weight supported on handling does not minimise stress induced by being caught by the tail^8,9^. Hurst & West^8^ suggested that aversion to being picked up by the tail probably stems from an ancestral imperative to avoid capture when fleeing from predators. For tunnel or cup handling, animals are approached from the front or side and the animal plays an active role in the process by stepping on to the hand or into the tunnel (even though this is enforced by the handler), with the handling device in clear view. By contrast, tail capture involves the hand approaching from behind followed by physical restraint in which the animal plays no active role; such complete lack of control over events, and inability to interact with and influence the process, can have very negative impacts on stress and anxiety^2,33^. It is also possible that being picked up by the tail causes some physical discomfort or pain. However, Miller & Leach^34^ found no difference in pain grimace scores between tail and tunnel handled mice. Further, as in the present study, procedures that are recognised to cause some discomfort or pain, such as injection, gavage, tattooing and ear tagging, do not induce aversion to handling if mice are picked up in a handling tunnel for the procedure^13,16^. This suggests that the strong handling aversion and anxiety induced by tail handling is a specific response to being captured and lifted by the tail rather than a more generic response to experiencing pain or discomfort when handled.

Although prolonged holding was not required to reduce handling aversion and anxiety, frequent handling was important for familiarising mice to being cupped on the open hand. Experience of only a small number of cup handling events (4–5) reduced anxiety but made little improvement in the willingness of mice to interact with the handler above those picked up by tail. However, this improved markedly with additional experience of brief cup handling between cage cleans. The much slower familiarisation of mice to accept cup handling compared to tunnel handling is consistent with previous findings, particularly in more anxious strains^8^. The number of handling events required to induce a positive response to cup handling also appears to vary between strains and the lighting phase when animals are handled, as Hurst & West^8^ found some improved interaction after just 5 daily handling events in ICR(CD-1) mice, and after the same number when BALB/c and C57BL/6 mice were handled during the light rather than the dark period. Tunnel handling results in a much more rapid and consistent positive response even from the outset^8,9,12,13^. As we show here, brief experience during cage cleaning appears to be sufficient to familiarise mice with the tunnel method. Brief tunnel handling also resulted in the most reliable responses in a behavioural test of stimulus investigation and recognition^11^, though few studies have directly compared tunnel and cup handling. In the current study, we assessed the willingness of animals to voluntarily approach the handling device, or to avoid being picked up when themselves approached by the handler (prolonged voluntary interaction corresponding negatively with handling avoidance). This required testing tail and cup handled mice with a gloved hand, but tunnel handled mice with a gloved hand holding the animals’ home cage tunnel. The area of cage occupied when a whole gloved hand or a tunnel (with hand held above) are inserted into the cage is very similar (see movies in reference 8), thus the considerably greater voluntary interaction with tunnels is not simply because mice are more likely to be in close proximity to a hand-held tunnel by chance. Further, the much greater willingness of tunnel-handled mice to approach their handling device is not simply because mice are more willing to approach a tunnel than the hand regardless of their handling experience. Hurst & West^8^ checked that mice familiar with tunnel handling also respond positively to a gloved hand alone, while Roughan & Sevenoaks^16^ tested the response of both tunnel and tail handled mice only towards a gloved hand. Both studies found substantially greater willingness to approach and interact with the gloved hand among mice familiarised with tunnel handling compared to those picked up by tail. Differences between methods in the amount of time spent in interaction with the hand were very similar to those observed in the current study.

Our findings have important implications from a practical perspective. First, the handling that mice experience during background husbandry has an important influence on their general anxiety and the threat they perceive when approached by people, even though such handling may be brief and relatively infrequent. The difference between tunnel and tail handled animals is particularly dramatic, with those picked up by tunnel actively seeking interaction and showing much more bold investigation when placed in a novel arena, while those picked up by tail show strong aversion and much greater caution in novel environments. Secondly, lengthy handling is not required to tame mice to accept handling and to actively seek human contact if an appropriate non-aversive method is used, such as the tunnel or cupping methods developed in our laboratory. As mice can be picked up just as quickly in a tunnel or scooped in the hand as picked up by the tail once handlers are skilled in these methods, it should be practicable to use these non-aversive methods instead of aversive tail handling for routine husbandry in most cases, as well as for experimental procedures. This will greatly reduce anxiety and stress responses to background handling, removing a confound that is very difficult to control across experiments and laboratories. Importantly, moving to non-aversive methods to pick up mice is a refinement that clearly has a substantial impact on animals and that avoids inducing stress unnecessarily, in line with ethical principles of animal use. While cupping mice on the open hand provides a clear refinement over tail handling, evidence so far suggests that using a handling tunnel where possible is the best method until mice have learned that being picked up is not threatening. As mice familiarised with tunnel handling also respond positively to a gloved hand alone^8,16^, and will readily sit on the hand once familiarised with being picked up in a tunnel^8^, prior tunnel handling can help to familiarise mice to being cupped on the open hand so that either method can then be used. As we and others have shown^13^, mice attempt to avoid capture by tail and are harder to handle than those picked up in a tunnel or familiarised with cupping. Using non-aversive methods will reduce the time and skill needed for routine handling, which is particularly important during husbandry procedures such as cage cleaning when large number of animals need to be handled efficiently.

Previous studies have shown that handling and restraint can induce much of the stress involved in routine laboratory procedures^35–37^, but it is usually assumed that this is largely a response to physical restraint. However, mice first familiarised with tunnel handling still interact very readily with the handler after experiencing restraint. This suggests that they did not learn to associate the tunnel with a negative experience that should be avoided. Most surprisingly, we found no difference in either willingness to interact with the handler or anxiety after mice were repeatedly immobilised and injected subcutaneously compared to those just picked up briefly. Instead, the method used to pick up mice prior to any procedure caused substantial differences in anxiety and willingness to interact, whether mice experienced the injection procedure or not. This indicates that the method used to pick up mice in the first place is much more important in influencing their anxiety and assessment of threat than experience of immobilisation and an injection that potentially induces pain. This is consistent with the findings of Nakamura & Suzuki^13^ and Roughan & Sevenoaks^16^, who compared the responses of tail versus tunnel handled mice after experience of various procedures (oral gavage, intraperitoneal injection, tattooing or ear tagging). Although these studies did not compare mice that underwent procedures with those that did not as we have done here, they found strong willingness to interact with the handler and low anxiety among tunnel handled mice despite experience of procedures thought to be stressful, while those picked up by tail showed aversion to the handler, stress during handling and high anxiety. The overriding effect of the method used to pick up mice over the procedure itself is some of the strongest proof yet that using a non-aversive method to routinely pick up mice has a substantial beneficial impact on their welfare, as well as reducing the likely confounding effects of handling stress on scientific research. Even very brief handling by the tail at cage cleaning has substantial negative effects and should be avoided whenever possible.

## Methods

Most of the procedures involved in this study were non-invasive behavioural tests. Animal use and care was in accordance with EU directive 2010/63/EU and UK Home Office code of practice for the housing and care of animals bred, supplied and used for scientific purposes. Response to subcutaneous injection was carried out under UK Home Office licence under the Animals in Scientific Procedures Act 1986 (authorisations PPL 40/3492 and PIL 40/10515). The University of Liverpool Animal Welfare Committee approved the work.

We conducted three separate experiments. Experiment 1 examined the effects of hold duration and handling method, and also tested response to scruff restraint. Experiment 2 investigated the effect of handling frequency and method when handling was brief. Experiment 3 examined the response to subcutaneous injection among mice familiarised with brief handling by tunnel or tail. Treatment groups, handling acclimation, animal characteristics and tests in each experiment are summarised in Supplementary Table 1, with methods provided in detail below. Throughout, animals were randomly allocated to each experimental group using a random numbers table, and the order of testing and animal handling was determined randomly using a random numbers generator, which identified the cage number where testing was to be initiated on each day. Behaviour was recorded on DVD and subsequently transcribed using an event timing program developed at the University of Liverpool by R. J. Beynon. All behavioural tests and handling were carried out during the active dark phase of the light cycle under dim red lights by KG. Our previous research has shown that responses to the different handling methods used in this study (voluntary interaction, stress during handling and anxiety) are very similar among mice handled during the light or dark phases of the light cycle^8^. Others have also found similar differences in response when handling mice during the light phase^10,13^.

### Experiment 1: Hold duration and handling method

#### Subjects and prior experience

C57BL/6JOla/Hsd mice (n = 80 mice per sex) were acquired at 3–4 weeks of age from Harlan UK. Throughout, mice were housed in single sex pairs in 43 × 11.5 × 12 cm cages (M3, North Kent Plastics Rochester, UK) on Corn Cob Absorb 10/14 substrate (IPS Product Supplies Ltd, London, UK) and provided with both water and food (lab diet 5002 certified rodent diet, Purina Mills) *ad-libitum*. All cages were enriched with paper wool nesting material (IPS Product Supplies Ltd) and a clear acrylic tunnel (50 mm wide and 150 mm long). Animals were kept on a reversed 12 h light/dark schedule (lights on 8 pm–8 am). All testing was carried out within the first four hours of the active dark phase under dim red light.

Each cage was randomly assigned to handling by one of three methods during the experiment: tail, home tunnel or cupping on the open hand (n = 16 cages per sex for home tunnel and cup methods; n = 8 cages per sex for tail). For three weeks from arrival until the start of the experiment (pre-conditioning phase), all mice assigned to tail were handled by tail at weekly cage cleaning. To assess whether prior experience of tail or non-aversive handling during routine husbandry had any subsequent influence, cages assigned to home tunnel or cupping were handled either by the same assigned method during the short pre-conditioning phase or by tail handling (n = 8 cages per sex per handling group, randomly allocated). However, as the handling method experienced during this brief period of background husbandry did not significantly alter any responses to the 5 days familiarisation with tunnel or cup methods addressed in this experiment, we have pooled mice regardless of their handling prior to the start of the experiment. Throughout, all handling was carried out by a single female experimenter (KG). To facilitate individual identification of cagemates on testing, two weeks after arrival mice (5–6 weeks old) were marked with hair dye on the shoulder or rump regions (B-blonde, cream peroxide 12%®), where they were handled by their assigned method. Mice were supervised during fur dyeing (returned to cages for up to 10 min), then dye washed out thoroughly to ensure no product residues were left on the animal (see Gouveia and Hurst^9^ for full details of the procedure). Sample sizes within each group were based on the number required to see relatively small differences in elevated plus maze and open field tests of anxiety.

#### Manipulation of hold duration

Once mice were 7–8 weeks old, mice in the same cage were picked up individually by their assigned method (tail, tunnel or tail) and held above their home cage for 2, 10, 30 or 60 s for five daily handling sessions (n = 8 mice for each handling method × duration × pre-conditioning combination; sample sizes within each group were based on the number required to see relatively small differences in elevated plus maze and open field tests of anxiety in a factorial design, assuming handling treatments had similar effects in males and females as previously observed^8,9,13^). For tail handling, the base of the tail was grasped between thumb and forefinger and mice were lifted onto the back of the experimenter’s gloved hand to support their weight during holding. This enabled us to standardize handling procedures for all mice picked up by this method, and ensured that mice undergoing more prolonged handling (up to 60 s) were not suspended by the tail. Tunnel handled mice were encouraged into their home cage tunnel with the other gloved hand approaching the animal gently from behind and lifted in the tunnel. Cupping was carried out by scooping the animal with both hands. In the first handling session, cupped mice were caught and held loosely between closed hands for up to 20 s to allow them to settle in the hand. In subsequent sessions, cupped mice were scooped and held above the cage on open hands and confined between hands only if necessary (until attempts to escape declined). For all three methods, mice assigned to 60 s daily handling were picked up twice and held for 30 s on each session (replicating the handling procedure used in previous studies^8–10,12,13^).

#### Voluntary interaction with the handler

We measured willingness to interact with the handler in anticipation of handling in a 60 s test carried out immediately before and after handling in the first and fifth daily handling session (as described in Hurst & West^8^). Briefly, the handler stood motionless in front of the home cage for 60 s, whilst holding the handling device in the front half of the home cage (a gloved hand for tail and cup methods, or hand holding the home cage tunnel for the tunnel method). We recorded total time spent in voluntary interaction with the handling device by each mouse (summed time sniffing with nose less than 0.5 cm from the handling device, front paws on handling device; climbing with all four paws on handling device, peeking with front paws in tunnel, all four paws inside tunnel, and chewing the glove or tunnel).

#### Open field test

Two days following the fifth daily handling session, each mouse was tested individually for 5 min in an open field, a well-validated test for measuring exploratory behaviour and anxiety in rodents^38,39^. Open field testing relies on the animal’s motivation to explore the central area of a test arena away from the side walls, where animals would be at greater risk of predation in the wild. High anxiety is evident by a high degree of thigmotaxis and avoidance of the central open area^38^. Mice were tested in a white laminated MDF (medium-density fibre board) arena measuring 70 × 60 × 55 cm high. Black PVC electric tape on the floor divided the arena into central (10 × 10 cm), middle (54 × 44 cm), and edge zones (3 cm from wall of the arena). For testing, the animal was delivered to the centre of the arena by its assigned handling method. Tail-handled mice were lifted by the tail but not supported on the hand as customary for short lifts^21^. Latency to first visit the arena edge (all four paws in the edge zone), time spent in central zone, number of visits to central zone, and the number of stretch attend postures (animal stretches forward with front half of body but retreats to its original position without moving hind feet^26,27^) were recorded as validated measures of anxiety in mice. Mice in the same cage were tested sequentially and the arena cleaned between animals using diluted Teepol® detergent and dried with a paper towel. The first mouse to be tested in each cage was temporarily placed in an empty holding cage after testing to minimise disruption of cagemate behaviour.

#### Repeated scruff restraint

Mice aged 8–9 weeks were restrained by the scruff once a day for three consecutive days. Animals were picked up by their assigned method (tail, tunnel or cup), placed onto their cage wire lid while gently restrained by the tail, and then immobilised by grasping the loose skin on the back of the neck (scruff) between thumb and forefinger. The mouse was lifted onto its back and held immobilised in the experimenter’s hand for 10 s before being returned to its home cage. Voluntary interaction with the handler (see procedure above) was assessed immediately before and 60 s after the first and third scruff restraint, and again 24 h following the third restraint when mice were picked up by their assigned method.

#### Elevated plus maze test

Two days following the third experience of scruff restraint, anxiety was assessed in a 5 min elevated plus maze test. The white plastic maze consisted of two open and two closed arms (all 30 × 5 cm with 15 cm high walls on the two closed arms), elevated 57 cm above the ground. Mice were delivered to the centre of the arena by their familiar handling method, facing an open arm. Anxiety in this test is evident in reduced exploration of the open arms and a high frequency of protected stretch attend postures (when the animal stretches forward into an open arm with the front half of its body but then retracts back into the protected centre or closed arms of the maze^26,27^). We recorded the frequency of protected stretch attend postures, the number of entries and time spent in the open arms, the number of entries and time spent in the closed arms of the maze and in the central hub. The maze was cleaned between animals using diluted Teepol® detergent and dried with a paper towel. The first mouse to be tested in each cage was temporarily placed in an empty holding cage after testing to minimise disruption of cagemate behaviour.

### Experiment 2: Frequency of handling using different methods

#### Subjects, housing and prior experience

BALB/cOlaHsd mice (n = 48 mice per sex) were acquired at 3–4 weeks of age from Harlan UK. Throughout, mice were housed in single sex pairs in 43 × 11.5 × 12 cm cages (M3, North Kent Plastics Rochester, UK) on Corn Cob Absorb 10/14 substrate (IPS Product Supplies Ltd, London, UK) and provided with both water and food (lab diet 5002 certified rodent diet, Purina Mills) *ad-libitum*. All cages were enriched with paper wool nesting material (IPS Product Supplies Ltd) and a clear acrylic tunnel (50 mm wide and 150 mm long). Animals were kept on a reversed 12 h light/dark schedule (lights on 8pm-8am). On arrival all mice were initially handled by tail to move them into home cages. Cages were cleaned out fortnightly. Three days before their first cage change (mice 5–6 weeks old), mice were individually marked using hair dye on the shoulder or rump regions (Clairol Natural Black®) to enable distinction between cagemates; all animals were handled briefly by the tail for this procedure.

#### Handling treatments

Cages were assigned randomly to one of six handling treatments starting at the first fortnightly cage clean: by tail at cage cleaning only or with supplementary daily handling; by home cage tunnel at cage cleaning only or with supplementary daily handling; by cupping at cage cleaning only or with supplementary daily handling (n = 8 mice per sex in each handling treatment). Animals were held for only a brief period during handling (approximately 2 s, equivalent to the time taken to transfer them between cages). For tail handling, mice were lifted by the base of the tail without body weight support, replicating the short 2 s lifts used in anxiety testing in Experiment 1. All animals were handled using their assigned method only during cage cleaning for the first four cage cleans. To test whether daily handling improved behavioural responses, mice in supplementary daily handling groups were briefly picked up by their assigned method for approximately 2 s and returned to their home cage for nine daily handling sessions between the fourth and fifth cage cleans.

#### Voluntary interaction with the handler

Willingness to interact voluntarily with the handler in a 60 s test was assessed immediately before and after handling at the first, fourth and fifth cage clean^8^. This followed the same procedure as experiment 1 except that the post-handling interaction test was carried out in a clean cage.

In addition to this, we also tested whether animals were reluctant to be handled when approached by the handler using the assigned method as if to capture them, assessed immediately after the post-handling interaction test in the clean cage. Each mouse was tested individually after transfer back to their empty home cage to ensure that their behaviour was not influenced by the presence of the cagemate. A mark was placed across the top of each test cage delimiting the front (closest to the handler) and back half of the cage. The animal was placed at the back of the cage facing the experimenter. Tunnel-handled mice were guided towards the tunnel entrance in the front half of the cage; reluctance was scored if the animal turned its head away from the tunnel and attempted to escape when it reached the tunnel entrance, but not if it entered the tunnel voluntarily. For tail handling, mice were gently touched on the back and the hand held at the back of the cage for 2 s, while for cup handling mice were approached from the side and the hand held in position for 2 s. If the animal immediately crossed to the front half of the cage without pausing it was scored as reluctant to be handled, but not if it paused for at least 1 s on its way to the front of the cage. For all methods, if the animal froze when approached by the handler, the test was repeated. Following testing, all animals were subsequently caught by their assigned method and returned to their home cage.

#### Elevated plus maze test

Two days after the fifth cage clean, all mice were tested in an elevated plus maze test, delivered to the maze by their assigned handling method. The procedure and measures were the same as in Experiment 1.

### Experiment 3: Repeated subcutaneous injection

#### Subjects, housing and prior experience

BALB/cOlaHsd mice (n = 80 females) were acquired at 3–4 weeks of age from Harlan UK. Housing and husbandry were the same as in Experiment 2, except that mice were individually marked using hair dye on the shoulder or rump regions (Clairol Natural Black®) at 8–9 weeks of age. To avoid prior taming effects of non-aversive handling before experimental procedures began, and in line with common husbandry practice at the time of the experiment, all mice were picked up and transferred between cages by tail during fortnightly cage cleans. Husbandry, handling and injection treatments were all carried out by the same female experimenter as in the previous experiments (KG).

#### Handling and injection treatments

Cages were assigned randomly to one of four treatment groups: tail handling with subcutaneous saline injection; tail handling only; tunnel handling with subcutaneous saline injection; tunnel handling only (n = 20 mice in 10 cages per group; sample sizes were based on moderate effect sizes and variance expected for anxiety measures in the modified open field test (see Gouveia & Hurst^11^), where there might be an interaction between handling method and experience of injection). At age 13–14 weeks, mice were picked up briefly (approximately 2 s) by their assigned handling method on 10 days over a two week period, previously shown sufficient to familiarise mice with non-aversive tunnel handling and overcome any aversion and anxiety induced by handling experience prior to the experiment^11^.

Two days following the 10^th^ daily handling session, mice were picked up by their assigned method. Those in the two injection groups were placed on top of a wire cage lid, restrained by the scruff and 0.2 ml sterile saline (Aqupharm™ No. 1) injected subcutaneously on the right flank using a 25 G needle. Mice were then placed in a clean empty holding cage for 5 min before being tested in a modified open field arena. Mice in handling only groups were picked up by their assigned method and placed directly in a clean holding cage for 5 min prior to testing.

To assess response to the experience of repeated subcutaneous injection, mice in the two injection groups were given a series of four daily saline injections starting six weeks after the initial injection, while mice in handling only groups were just picked up by their assigned method. The delay allowed us to assess responses to the first injection procedure before continuing. The injection site was alternated between left and right flanks. Following the last repeat injection (or control pick up), mice were again delivered to an empty holding cage for 5 min prior to a repeat modified open field test. On the other days, mice were replaced directly in their home cages.

#### Modified open field test

To assess anxiety immediately after experience of injection (1^st^ and 5^th^) or control pick up, mice were placed into an open arena (70 × 60 × 55 cm white laminated MDF) modified by the presence of a male mouse urine stimulus in the centre of the arena. This modified test measures response to a rewarding stimulus as well as willingness to explore a novel arena, which can provide valuable insight into an animal’s emotional state^12,40,41^. The presence of a novel urine stimulus also helps to maintain the novelty of the test when mice need to be tested more than once. Male mouse urine is an attractive and highly rewarding stimulus to adult female mice across the oestrous cycle that should stimulate close contact investigation and time spent near the stimulus, even when it is placed in the open central area of an arena^11,42–44^. Urine samples were obtained from four unrelated sexually mature male wild-stock mice, held by the scruff over clean Eppendorf tubes, and pooled to provide an homogeneous stimulus over all trials (separate pools used for first and second test). 10 μl urine was streaked on a microscope slide stuck to a plastic tile (15 × 15 cm) placed in the centre of the arena. Mice were delivered to the periphery of the arena, halfway along one side wall, by their assigned method (tail handled mice were moved without body support following common practice for short lifts) for a 5 min test, recorded on DVD. The delivery location was marked with a piece of black electrical tape on the top arena edge to ensure that all animals were delivered to the same location for testing. For assessment of behavioural responses, an acetate sheet was placed over a computer screen to delimit the different areas of the test (centre, middle, periphery and corners). We assessed the number of lines crossed (by all four paws) as mice moved between the different areas of the arena as a measure of movement around the arena, latency to first enter the central zone, frequency of stretch attend postures, frequency of visits to the urine stimulus tile and time spent sniffing the urine. The maze was cleaned between animals using diluted Teepol® detergent and dried with a paper towel. The first mouse to be tested in each cage was temporarily placed in an empty holding cage after testing to minimise disruption of cagemate behaviour.

#### Voluntary interaction with the handler

Willingness to interact voluntarily with the handler was assessed in a 60 s test conducted both immediately before and after (i) the 10^th^ handling session, (ii) the first injection or control pick up, and (iii) the fifth injection or control pick up. The procedure was the same as in Experiment 1. Following each post-handling interaction test, we tested the reluctance of mice to be handled, following the same procedure as Experiment 2.

### Data analysis

Statistical analyses were carried out using SPSS versions 24 and 25 (IBM software). All data plotted in figures are untransformed. Total time spent in voluntary interaction with the handler at each time point was averaged across the two mice in the same cage as the behaviour of animals tested together was not independent. The effects of handling method, hold duration and sex (Experiment 1), or handling method, frequency and sex (Experiment 2) were examined by univariate ANOVA at each time point, and compared between time points using repeated measures ANOVA (data log transformed when necessary to meet parametric assumptions, see footnotes to Tables). Planned contrasts compared non-aversive tunnel or cup handling to traditional tail handling in each analysis. Bonferroni post-hoc comparisons examined differences in response between different handling durations (Experiment 1) where handling duration had a significant effect. For each model, we checked the distribution of residuals using frequency histograms and normal Q-Q plots, together with Shapiro-Wilks tests to assess any major deviation from normality. All data are included in our models, but we confirmed that removal of any clear outliers did not change levels of statistical significance and thus were not biasing our analysis and conclusions. Where residuals showed some deviation from normality due to a few zero values, we also checked that all significant effects showed very similar levels of significance in simpler nonparametric analyses (three handling methods and four handling durations compared using Kruskal-Wallis tests; two handling frequencies and planned contrasts between tail versus tunnel and tail versus cup compared using Mann-Whitney tests). There was unequal variance in voluntary interaction between groups when data did not require log transformation (Experiment 2), but as F-tests are very robust to heteroscedasticity when group sizes are equal^45^ this did not cause a problem for our analysis. In experiment 3, nonparametric Mann-Whitney tests assessed the effects of handling method (tail versus tunnel) and treatment (restraint and injection versus control pick up) at each time point, while Friedman repeated measures tests assessed differences between time points for each treatment group. Contingency chi-squared tests compared the number of mice reluctant to be handled between treatment groups (Experiments 2 & 3).

Repeated measures ANOVAs examined the effects of handling method, duration or frequency, and sex on measures of anxiety in open field and elevated plus maze tests, with the mouse tested first or second from the same cage included as a within-subjects factor to take into account any lack of independence between mice housed together (Experiments 1 & 2). The distributions of residuals and any deviations from normality were checked as above for each model to ensure that the assumption of normality did not bias our analysis and conclusions. As there was a high degree of correlation between measures of anxiety in the modified open field test used in Experiment 3, principal components analysis was used to derive a single component of behaviour from the five measures taken from the modified open field test (see above). This explained 43% of variance in behaviour (weightings given to each component are given in the figure legend to Table 8). No other components achieved an eigenvalue greater than 1. We used mean responses per cage rather than separate data for each mouse in a repeated measures design because data for one mouse was missing for five cages (two mice were withdrawn from the experiment for health reasons and three because of technical errors in the test). A small number of data points missing in Experiments 1 and 2 were also due to technical errors during specific tests (see Supplementary File 1 for raw datasets).

## Supplementary information

Supplementary Information.

Supplementary Information 2
