# Supplementary material for: Improving the practicality of using non-aversive handling methods to reduce background stress and anxiety in laboratory mice
Source: Sci Rep. 2019 Dec 30;9:20305. doi: 10.1038/s41598-019-56860-7 (PMC6937263; doi:10.1038/s41598-019-56860-7)
Supplement: Supplementary file 1 — Supplementary Information. [file 41598_2019_56860_MOESM1_ESM.pdf]

Improving the practicality of using non-aversive handling methods to reduce background stress and anxiety in laboratory mice

Kelly Gouveia & Jane L. Hurst

Supplementary information

Supplementary Table 1. Overview of experiments

Experiment and stage	Treatment groups	Handling acclimation	Animal characteristics	Tests
Exp 1 stage 1: Effect of hold duration and handling method	Handling method: tail, tunnel, cup<br>Hold duration: 2, 10, 30, 60s<br>Sex: male, female	Daily handling by assigned method and hold duration for 5 days<br>Aged 7-8 weeks	C57BL/6JOla/Hsd<br>Males and females<br>Housed two per cage<br>n = 8 cages per hold duration for tunnel and cup methods<br>n = 4 cages per hold duration for tail method	Voluntary interaction immediately before & after handling on 1st and 5th daily handling sessions<br>Open field test two days after 5th handling session
Exp 1 stage 2: Response to scruff restraint	Handling method: tail, tunnel, cup<br>Hold duration in previous week: 2, 10, 30, 60s<br>Sex: male, female	Pick up by assigned method and 10s daily scruff restraint for 3 days<br>Aged 8-9 weeks	Same mice as above	Voluntary interaction immediately before & after 1st and 3rd scruff restraint<br>Elevated plus maze test two days after 3rd scruff restraint
Exp 2: Effect of handling frequency and method	Handling method: tail, tunnel, cup<br>Frequency: cage clean only versus cage clean plus supplementary handling<br>Sex: male, female	All mice: 2s at fortnightly cage clean by assigned method<br>Half received additional nine daily 2s lifts by assigned method between 4th and 5th cage cleans<br>From age 5-6 weeks	BALB/cOlaHsd<br>Males and females<br>Housed two per cage<br>n = 8 cages per frequency and handling method	Voluntary interaction immediately before & after 1st, 4th and 5th cage clean<br>Reluctance to be handled after 1st, 4th and 5th cage clean<br>Elevated plus maze test two days after 5th cage clean
Exp 3: Subcutaneous injection and handling method	Handling method: tail, tunnel<br>Procedure: scruff restraint + subcutaneous saline injection versus control lift only	2s daily by assigned handling method for 10 days<br>From age 13-14 weeks<br>1st injection or control lift aged 15-16 weeks<br>2nd – 5th injection (daily injections) or control lift aged 21-22 weeks	BALB/cOlaHsd<br>Females<br>Housed two per cage<br>n = 10 cages per method and procedure group	Voluntary interaction immediately before & after 10th handling session, 1st injection or control lift, 5th injection or control lift<br>Modified open field test after 1st and 5th injection or control lift
